# Special Issue “Diseases of the Salivary Glands-Part II”

**DOI:** 10.3390/jcm11195567

**Published:** 2022-09-22

**Authors:** Margherita Sisto

**Affiliations:** Department of Basic Medical Sciences, Neurosciences and Sense Organs, Section of Human Anatomy and Histology, University of Bari Medical School, 70124 Bari, Italy; margherita.sisto@uniba.it

This Special Issue, “Diseases of Salivary Gland-Part II”, was born as a continuation of the volume “Diseases of the Salivary Gland”, published, with great success, in 2021 in the prestigious *Journal of Clinical Medicine* (*JCM*) (https://www.mdpi.com/journal/jcm/special_issues/Salivary_Glands, accessed on 3 April 2021). The management of salivary gland (SGs) disorders encompasses a broad array of diseases, both benign and malignant, and the great international interest in this topic has led to the publication of the second part of the Special Issue, thusly entitled “Diseases of the Salivary Glands-Part II”, which reflects the diverse nature of SG dysfunction and is focused on various topics ranging from diagnosis and therapeutic implications to the study of the molecular mechanisms underlying autoimmune and neoplastic diseases of the salivary glands.

The first part of this Special Issue concerns research articles focused on new discoveries in the diagnostic field of SG diseases [1,2,3,4,5,6] and on the identification of molecular mechanisms that could correlate the state of chronic inflammation that characterizes autoimmune SG diseases with the dysfunction of the glands themselves [1,2,3,4,5,6]. SGs are of the utmost importance for maintaining the health of the oral cavity and carrying out physiological functions such as mastication, the protection of teeth, the perception of taste, and speech. José Mário Matos-Sousa and colleagues from the Federal University of Pará, Brazil, examined whether fluoride—known to be effective in preventing and controlling caries—damages SGs by inducing biochemical and proteomic changes; interestingly, the research group demonstrated that fluoride does not cause any morphological or biochemical changes in the SGs and so reinforced the effectiveness of a low-dose fluoride treatment for the maintenance of cavity homeostasis [1]. Moreover, a comparative study from Taiwanese researchers comparing the effectiveness of hydrogen peroxide and adrenaline during tonsillectomy was included, which aimed at safeguarding the integrity of the SGs of the entire oral mucosa [4]. Among the research papers, a portion of this Special Issue is dedicated to the study of the molecular mechanisms underlying Sjögren’s syndrome (SS), a chronic inflammatory disease, with a varied and still partly unknown etiology, which leads to the dysfunction of the SGs and xerostomia. The Special Issue collects the most recent discoveries made by the Sisto and Lisi group that correlate the inflammatory degree of SGs with the Epithelial to Mesenchymal Transition (EMT) program activation in SS, underlining the possibility of a fibrotic evolution of the glandular tissue [2]. Furthermore, a recent study of Campagna et al., resulting from an international collaboration between Italy and Colombia, investigated and clarified the role of T lymphocytes in SS, universally accepted as actors in the pathogenesis of SS [3]; in addition, an interesting study by Gupta and colleagues from the University of Florida enriched this Special Issue by contributing an article describing the possibility of mapping the epitopes of pathogenic autoantigens on SS leukocytes [5]. Other authors, such as the group of Micaela F. Beckman and Farah and Jean-Luc Mougeot (conducted at Carolinas Medical Center, Charlotte, NC, USA), analyzed data relating to the expression of specific antigens associated with SS in order to identify new therapeutic targets for xerostomia [6]. The etiology of SS remains poorly understood; however, as evidenced by the section of this Special Issue dedicated to reviews [7,8,9,10], the findings are becoming increasingly interesting and lately they correlate SS with the activation of an EMT program that could explain the altered function of the SGs in SS with a reduction in saliva secretion. It is interesting to underline that, up to now, the EMT is a process related to neoplastic transformation, as also reported for SG tumors by Yuka Matsumiya-Matsumoto from Osaka University [8]; however, in recent years, vast amounts of evidence have been collected that highlights the activation of the EMT even in situations of chronic inflammation, similar to that which occurs in autoimmune diseases [7,9,10].

Given the scientific importance of the articles collected, I am thrilled to reiterate that this Special Issue aims to provide insights into SGs diseases and summarizes the current knowledge of underlying pathophysiological mechanisms, yielding surprising results.

## References

[1] Matos-Sousa J., Bittencourt L., Ferreira M., dos Santos V., Balbinot K., Alves-Júnior S., Pinheiro J., Charone S., Pessan J., Lima R. (2022). Fluoride Exposure and Salivary Glands: How Is Glandular Morphology Susceptible to Long-Term Exposure? A Preclinical Study. J. Clin. Med..

[2] Sisto M., Ribatti D., Ingravallo G., Lisi S. (2022). The Expression of Follistatin-Like 1 Protein Is Associated with the Activation of the EMT Program in Sjögren’s Syndrome. J. Clin. Med..

[3] Campagna G., Anzola L., Varani M., Lauri C., Gentiloni Silveri G., Chiurchioni L., Spinelli F., Priori R., Conti F., Signore A. (2022). Imaging Activated-T-Lymphocytes in the Salivary Glands of Patients with Sjögren’s Syndrome by 99mTc-Interleukin-2: Diagnostic and Therapeutic Implications. J. Clin. Med..

[4] Hsieh C., Hsu C., Wu H., Sun C. (2022). Comparison Benefit between Hydrogen Peroxide and Adrenaline in Tonsillectomy: A Randomized Controlled Study. J. Clin. Med..

[5] Gupta S., Li D., Ostrov D., Nguyen C. (2022). Epitope Mapping of Pathogenic Autoantigens on Sjögren’s Syndrome-Susceptible Human Leukocyte Antigens Using In Silico Techniques. J. Clin. Med..

[6] Beckman M., Brennan E., Igba C., Brennan M., Mougeot F., Mougeot J. (2022). A Computational Text Mining-Guided Meta-Analysis Approach to Identify Potential Xerostomia Drug Targets. J. Clin. Med..

[7] Kelly A., Nelson R., Sara R., Alberto S. (2022). Sjögren Syndrome: New Insights in the Pathogenesis and Role of Nuclear Medicine. J. Clin. Med..

[8] Matsumiya-Matsumoto Y., Morita Y., Uzawa N. (2022). Pleomorphic Adenoma of the Salivary Glands and Epithelial–Mesenchymal Transition. J. Clin. Med..

[9] Sisto M., Ribatti D., Lisi S. (2022). Sjögren’s Syndrome-Related Organs Fibrosis: Hypotheses and Realities. J. Clin. Med..

[10] Sisto M., Ribatti D., Lisi S. (2022). E-Cadherin Signaling in Salivary Gland Development and Autoimmunity. J. Clin. Med..

